# Targeted next generation sequencing of RB1 gene for the molecular diagnosis of Retinoblastoma

**DOI:** 10.1186/s12885-015-1340-8

**Published:** 2015-04-28

**Authors:** Bharanidharan Devarajan, Logambiga Prakash, Thirumalai Raj Kannan, Aloysius A Abraham, Usha Kim, Veerappan Muthukkaruppan, Ayyasamy Vanniarajan

**Affiliations:** 1 Department of Bioinformatics, Aravind Medical Research Foundation, Madurai, India; 2 Department of Molecular Genetics, Aravind Medical Research Foundation, Madurai, India; 3 Department of Orbit, Oculoplasty and Oncology, Aravind Eye Hospital, Madurai, India; 4 Advisor-Research, Aravind Medical Research Foundation, Madurai, India

**Keywords:** Retinoblastoma, Targeted next generation sequencing, Molecular diagnosis

## Abstract

**Background:**

The spectrum of *RB1*gene mutations in Retinoblastoma (RB) patients and the necessity of multiple traditional methods for complete variant analysis make the molecular diagnosis a cumbersome, labor-intensive and time-consuming process. Here, we have used targeted next generation sequencing (NGS) approach with in-house analysis pipeline to explore its potential for the molecular diagnosis of RB.

**Methods:**

Thirty-three patients with RB and their family members were selected randomly. DNA from patient blood and/or tumor was used for *RB1* gene targeted sequencing. The raw reads were obtained from Illumina Miseq. An in-house bioinformatics pipeline was developed to detect both single nucleotide variants (SNVs) and small insertions/deletions (InDels) and to distinguish between somatic and germline mutations. In addition, ExomeCNV and Cn. MOPS were used to detect copy number variations (CNVs). The pathogenic variants were identified with stringent criteria, and were further confirmed by conventional methods and cosegregation in families.

**Results:**

Using our approach, an array of pathogenic variants including SNVs, InDels and CNVs were detected in 85% of patients. Among the variants detected, 63% were germline and 37% were somatic. Interestingly, nine novel pathogenic variants (33%) were also detected in our study.

**Conclusions:**

We demonstrated for the first time that targeted NGS is an efficient approach for the identification of wide spectrum of pathogenic variants in RB patients. This study is helpful for the molecular diagnosis of RB in a comprehensive and time-efficient manner.

## Background

Retinoblastoma (RB, OMIM#180200), the most common pediatric eye tumor in the retina is initiated by inactivating biallelic variants of *RB1* gene [1]*.* Retinoblastoma occurs in hereditary and non-hereditary forms, with most bilateral and some unilateral RB cases being hereditary. The non-heritable form predominantly leads to unilateral tumors where in both variants have occurred in somatic cells and are not transmitted [2]. It is essential to identify and distinguish the germline and somatic variations in *RB1* for predicting the accurate risk of RB in future siblings and offsprings. The retinoblastoma susceptibility gene, *RB1* (Genbank accession number L11910.1; NCBI RefSeq NM_000321.2) is located on chromosome 13q14.2 and is composed of 27 exons distributed along 183 kb of genomic sequence. A wide spectrum of heterogeneous *RB1* gene variants that includes – single nucleotide variations (SNVs), small insertions/deletions (InDels) and structural variations (SVs) had been reported in RB patients [3]. Some of the variants such as nonsense and frameshift are associated with bilateral RB, while other types have unilateral RB or milder phenotypic expression [4].

Predictive genetic testing of RB can help to save the vision and avoid unnecessary (and invasive) eye examinations for patients and their close relatives in a cost effective manner. Currently, the routine procedure for genetic testing of *RB1* involves multiple methods of mutation detection in the coding regions and intron-exon boundaries using Sanger sequencing, and deletion/duplication analysis by genotyping methods such as multiplex ligation-dependent probe amplification (MLPA), quantitative multiplex PCR (QMPCR) [5]. The major limitations of Sanger sequencing are the extended time taken for screening all 27 exons individually and limited data (2X) generated from the sequencing runs. Thus, identifying the spectrum of heterogeneous variants in *RB1* gene makes the molecular diagnosis of RB challenging and time-consuming.

Accurate identification of *RB1* pathogenic variants in a reduced time is very important for diagnosis, confirmation, genetic counseling, risk assessment, and carrier screening of RB patients and their family members. Next Generation sequencing (NGS) has been found to be a time-efficient and accurate approach for the molecular diagnosis of simple to complex diseases including cancer [6-8]. Due to this improved efficiency, NGS has been widely used as diagnostic tool for retinal dystrophies [9-12]. In the present study, we have used targeted next generation sequencing approach with in-house bioinformatics pipeline for the molecular diagnosis of RB for the first time.

## Methods

### Clinical diagnosis and patient samples

A total of 21 families with bilateral RB and 12 families with unilateral RB were selected for this study (Table 1). The clinical diagnosis of RB was made by thorough clinical examination and radiological investigations (CT/MRI and USG B scan) along with Retcam imaging in Aravind Eye Hospital Madurai, India. Retinal examination was performed in family members to detect small scars/pigmentary changes, which are suggestive of regressed RB. The blood samples were collected from patients and family members. In addition, fresh tumor samples were collected from enucleated patient eyes. The present study was approved by the Institutional Ethics Committee of Aravind Medical Research Foundation, Madurai, India (Registration Number: ECR/182/Arvind/Inst/TN/2013). All the patient samples were collected after getting the informed consent from the families.

**Table 1:** **Clinical & family history of RB patients and samples selected for NGS**

Patient no	Age (months)/Sex	Laterality	Family history	Samples analysed by NGS
RB1	4/F	Bilateral	Father with regressed RB	Patient’s blood
RB2	1/F	Bilateral	Father with regressed RB	Patient’s blood
RB3	33/F	Bilateral	Nil	Patient’s blood
RB4	4/M	Bilateral	Mother and sibling affected with RB	Patient’s blood
RB5	0/F	Bilateral	Nil	Patient’s blood
RB6	31/F	Bilateral	Three siblings affected with RB	Patient’s blood
RB7	39/F	Bilateral	Father affected with RB	Patient’s and Father’s blood
RB8	44/M	Unilateral	Nil	Patient’s blood and tumor
RB9	7/F	Unilateral	Nil	Patient’s tumor
RB10	0/M	Unilateral	Nil	Patient’s tumor
RB11	5/F	Unilateral	One sibling affected with RB	Patient’s and Sibling’s blood
RB12	14/M	Unilateral	Nil	Patient’s blood and tumor
RB13	12/M	Bilateral	Nil	Patient’s blood
RB14	44/F	Bilateral	Father and sibling affected with RB	Patient’s blood
RB15	11/M	Bilateral	Father affected with RB	Patient’s blood
RB16	0/M	Bilateral	Nil	Patient’s blood
RB17	0/M	Bilateral	Father and sibling affected with RB	Patient’s blood
RB18	26/F	Bilateral	Nil	Patient’s blood
RB19	36/F	Bilateral	Nil	Patient’s blood
RB20	31/M	Bilateral	Nil	Patient’s blood
RB21	12/M	Unilateral	Nil	Patient’s tumor
RB22	18/F	Unilateral	Nil	Patient’s tumor
RB23	19/M	Bilateral	Father and sibling affected with RB	Patient’s blood
RB24	26/F	Bilateral	Nil	Patient’s blood
RB25	8/F	Bilateral	Nil	Patient’s blood and tumor
RB26	12/F	Bilateral	Nil	Patient’s blood
RB27	66/F	Bilateral	Nil	Patient’s blood
RB28	58/F	Unilateral	Nil	Patient’s tumor
RB29	28/M	Unilateral	Nil	Patient’s tumor
RB30	18/M	Unilateral	Nil	Patient’s tumor
RB31	88/M	Unilateral	Nil	Patient’s tumor
RB32	27/M	Unilateral	Nil	Patient’s tumor
RB33	3/M	Bilateral	Nil	Patient’s blood

The age at which first sign was detected is given in months. Laterality was confirmed by the clinical investigations and imaging (CT Scan/MRI, Ultrasound, Retcam). Family history was ascertained by three or four generation pedigree. NGS was performed on patient’s tumor wherever available. In three patients (RB8, RB12 and RB25), both blood and tumor samples were analyzed. In two families, affected members were also included for NGS along with patients (RB7 and RB11).

### DNA isolation

Genomic DNA was isolated from blood samples (2 ml for patients and 5 ml for parents) by salting out method [13] and tumor by QIAamp® DNA Mini Kit (Qiagen, Germantown, MD) following the manufacturer’s protocol. The quality and quantity of the DNA was checked by Nanodrop 1000 spectrophotometer (Thermo Scientific, Waltham, USA).

### Library preparation and targeted next generation sequencing

Targeted NGS was performed in total of 33 patients. Of those, 12 were tumor and 21 were blood samples. In three patients, tumor/blood matched pairs were included. In two families, the affected family members along with the patient were also analysed (Table 1). A Primer library was custom-designed to amplify 27 exons, exon/intron boundaries and promoter region of *RB1* gene using the Illumina Truseq custom Amplicon and Agilent SureSelect in-solution hybridization capture kits by the service provider (Scigenom, Kochi, India). Briefly, 2 μg of each genomic DNA was sheared into 100-500 bp fragments. Regions of interest were enriched using the above methods and libraries were prepared. The high sensitivity DNA chips were used in Agilent Bioanalyzer, to validate the enrichment process. Quantitative PCR was used to measure the quantity of the library before sequencing. Captured libraries were sequenced in a multiplexed fashion on Miseq with paired end run to obtain 2×150 bp reads with at least 100X depth of coverage. The coding region with <20X depth of coverage were covered by Sanger sequencing.

### SNV and InDel detection

**Figure 1:**
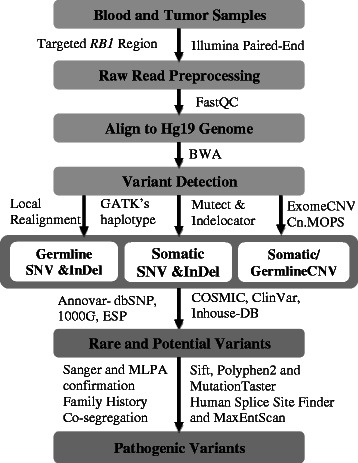
Analysis pipeline to identify pathogenic variants in tumor and blood samples from retinoblastoma patients.

Obtained raw sequence reads from Miseq were analysed using bioinformatics pipeline as shown in Figure 1. Data was quality filtered using fastQC tool [14]. The filtered reads were mapped to Hg19 reference sequence using Burrow-Wheeler Aligner (BWA version 0.7.5a-r405) [15]. Resulting BAM files were locally realigned using GenomeAnalysisTK-3.1-1 (GATK) Indel-realigner [16] tool to minimize the mismatches across the reads. GATK haplotype caller was performed to retrieve germline single nucleotide variants (SNVs) and small insertions/deletions (InDels) with phred score 20 and minimum depth 5 from all the samples. MuTect-1.1.4 [17] and GATK Indelocator tools were used to identify somatic SNVs and InDels from the tumor samples with blood matched control respectively. Wherever matched blood sample is not available, the blood sample with similar coverage was used. All the SNVs and InDels were subjected to identify rare and potential variants. The rare variants were identified using ANNOVAR [18] by filtering common variants with alternative allelic frequency higher than 1% based on 1000 Genomes project (http://www.1000genomes.org/data), dbSNP135 (http://www.ncbi.nlm.nih.gov//SNP) and ESP (http://evs.gs.washington.edu/EVS). Of those, non-synonymous/synonymous SNVs, coding InDels, and intronic variants that were less than 10 bp beyond the canonical splice site junction were selected. The potential variants were identified using ClinVar (http://www.ncbi.nlm.nih.gov/clinvar/), COSMIC (http://cancer.sanger.ac.uk/cosmic) and In-house (reported pathogenic variants) databases. The detection of germline SNVs and Indels was fully automated. Detected variants were further manually assessed with the help of IGV-2.3.25 viewer to avoid mapping errors.

### Copy number variation (CNV) detection

The locally realigned BAM files were used to detect CNV. ExomeCNV [19], a statistical tool that uses coverage and alternative allele frequencies to estimate CNV, was used to detect somatic CNVs from the tumor/blood pairs as described above. Whereas, Cn. MOPS [20], a read count based CNV caller was used to detect the germline CNVs in the blood samples. More than five blood samples with similar exon coverage were used to improve the sensitivity of Cn. MOPS. Log Ratios (LogR) ≥ ±1 were set for Deletion/Duplication analysis in both the tools; median LogR score were used for Cn. MOPS.

### Identification of pathogenic variants

In order to identify pathogenic variants, we used the following criteria. i) known pathogenic variants; ii) if not, variants that could give rise to premature protein termination, frameshift, canonical splice site alterations and large exonic deletions; iii) nonsynonymous SNVs if Sift [21], Polyphen2 [22] and MutationTaster [23] all suggested pathogenic, and iv) splice variants selected from both Human splice site finder [24] and MaxEntScan [25].

### Confirmation of variants by Sanger sequencing and MLPA

All the pathogenic variants were confirmed by Sanger sequencing, MLPA and cosegregation analysis in blood and tumor samples of patients, and blood samples of family members. PCR amplification of the corresponding exon around the variant site of the *RB1* gene was performed. Each 25 ul reaction contained 20 ng of genomic DNA, 10XPCR Buffer, 100 mM dNTPS, 10uM of each forward and reverse primer and 1U of Taq DNA polymerase (Sigma Aldrich, Missouri, USA) with cycling conditions and PCR primers described previously [26]. Cycle sequencing was performed using the BigDye Terminator kit version 3.1 and purified products were analyzed on a 3130 Genetic Analyzer (Life Technologies, USA). MLPA was performed with SALSA MLPA kit P047-*RB1* kit (MRC-Holland, Amsterdam, The Netherlands) according to manufacturer’s instructions. Fragment analysis was performed with Gene Mapper software (Life Technologies, USA) and data was analyzed using Coffalyser software (MRC, Holland) where DNA copy number ratios of tumor samples were computed using the matched blood sample. For genes targeted by multiple probes, copy number ratios were averaged. A threshold ratio of >1.3 denotes duplication and a ratio of <0.7 denotes deletion. Size fractionation was carried out by agarose gel electrophoresis to confirm deletions ranging from 10 to 30 bp.

### Targeted sequencing using Ion-Torrent Personal Genome machine (PGM)

In order to compare the data obtained from Miseq, eight patients were selected randomly for the cross platform comparison. Of those, six were blood from patients RB2, RB4, RB7, RB13, RB24, RB25 and two were tumor/blood matched pairs of samples from RB8 and RB12. Sequencing was performed with Ion-Torrent PGM at University of Pennsylvania, Philadelphia using the protocol as described [27]. Briefly, multiplex PCR was performed to generate the PCR fragments of all 27 exons of RB1 using Qiagen multiplex PCR kit. Ion Xpress Plus gDNA Fragment Library Preparation kit was used for shearing, adapter ligation and nick repair. Emulsion PCR was performed with One Touch2 system and enrichment with One Touch ES using 200 bp chemistry. Purification was performed at each step with Ampure beads (Agencourt) and quality was checked using the Bioanalyzer. The final enriched libraries were sequenced on Ion PGM with 318 chip. The sequence reads were aligned against the human RB1 genomic sequence [GenBank Accession L11910.1] and variant calling were made using Ion Torrent Suite (Life Technologies) as described [27]. The reads were automatically barcode-sorted followed by removal of the reads with low quality. The BAM and BAI files of Ion PGM runs were checked visually on IGV-2.3.25 viewer to avoid sequencing errors associated with homopolymer regions.

## Results

### Targeted sequencing characteristics

Thirty three patients with RB as shown in Table 1 from unrelated Indian families were selected for this study of targeted RB1 sequencing. Illumina Truseq Custom Amplicon was used for target amplification in 23 samples (RB1-RB23) and Agilent SureSelect enrichment method was used for other 10 samples (RB24-RB33). Paired end sequencing in Miseq covered nearly 3000 bases of RB1 encompassing 27 exons along with their flanking intron and promoter regions. The mean depth of coverage was found to be ~ 200X with Truseq and ~150X with SureSelect. The average sequencing coverage of the targeted regions was 98.0% in Truseq compared to 99.8% in SureSelect as exons 14 and 20 were not covered sufficiently (<20X) in Truseq. The missed regions were covered by Sanger sequencing. Therefore, complete coverage of all the target bases was ensured to provide high quality bases for sensitive and efficient variant detection. An automatic in-house variant calling pipeline was developed using freely available tools to detect germline SNVs and InDels for all the samples, wherein the tumor samples were checked for somatic SNVs and InDels. With stringent criteria, pathogenic SNVs and InDels were identified and patients with no pathogenic variants were further analysed for copy number variations (CNVs). ExomeCNV and Cn. MOPS were used to detect somatic and germline CNVs in tumor and blood samples respectively. All the pathogenic variants were further confirmed by conventional methods and cosegregation. Somatic events were re-confirmed by their absence in same patient blood sample.

### Identification of germline SNVs and InDels in RB patients

Blood samples of 21 bilateral (familial and sporadic) patients and one familial unilateral patient (Table 1), were analyzed to detect SNVs and InDels. Pathogenic variants were identified in 15 patients, of which eight were novel and seven were previously reported (Table 2). Surprisingly, all the reported pathogenic variants were found to be nonsense variants, resulting in premature protein termination. Five of them were shown to be *de novo* as only the patient had the mutation and not the family members, and remaining two were inherited from one of their parents (Table 1 and 2).

**Table 2:** **RB1 variants identified by targeted NGS in blood samples of RB patients**

Patient no	cDNA change	Amino acid change	Functional consequence	Cosegregation in family
RB1	**c.-212_-195del**		Promoter Deletion	Heterozygous Father
RB2	c.1399C > T	p.R467X	Premature Protein Termination	Heterozygous Father
RB4	**c.265-9 T > A**		Altered Splicing	Heterozygous Mother and Sibling
RB11	**c.46_74del**	**p.A16AfsX14**	Frameshift	Heterozygous Mother and three Siblings
RB13	c.751C > T	p.R251X	Premature Protein Termination	*De novo*
RB14	**c.2520 + 4 A > G**		Altered Splicing	Heterozygous Father
RB15	**c.2115_2118del**	**p.M705IfsX8**	Frameshift	Heterozygous Father
RB16	c.1363C > T	p.R455X	Premature Protein Termination	*De novo*
RB17	**c.1960 + 2 T > A**		Altered Splicing	Heterozygous Father and Sibling
RB18	**c.38_66del**	**p.A13AfsX17**	Frameshift	*De novo*
RB19	c.1399 C > T	p.R467X	Premature Protein Termination	Heterozygous Mother
RB24	**c.1961_1963del**	**p.654_655del**	Altered Splicing	*De novo*
RB25	c.1072C > T	p.R358X	Premature Protein Termination	*De novo*
RB26	c.521 T > A	p.L174X	Premature Protein Termination	*De novo*
RB27	c.160G > T	p.E54X	Premature Protein Termination	*De novo*

Novel variants are marked in bold. Cosegregation of the variants was confirmed by Sanger sequencing analysis of the variants in family members.

**Figure 2:**
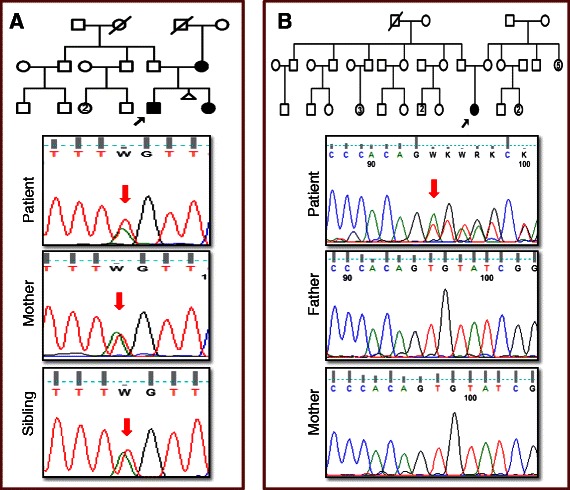
Confirmation of novel pathogenic splice variants. **(A)** Cosegregation of variants in the family was confirmed by Sanger sequencing of blood samples of Patient RB4, his mother and sibling, who had heterozygous c.265-9 T > A variant that created a new splice site acceptor. **(B)** Patient RB24 had a *de novo* heterozygous in-frame deletion of three bases identified at the start site of exon 20. Red arrows denote the variant.

The novel pathogenic variants either caused aberrant splicing or frameshift due to deletions. Four variants identified in patients RB4, RB14, RB17 and RB24 were found to affect splicing based on the HSF and MaxEntScan tools (Table 2). Patient RB4 was found to have a heterozygous c.265-9 T > A intronic variant at the upstream of acceptor splice site, which was predicted to activate a cryptic splice site (9 bases prior to exon 3) that may result in frameshift. In case of patient RB14 and RB17, heterozygous intronic variants near canonical splice sites might result in altered splicing. Three patients RB4, RB14 and RB17 had affected members in the family showing the same variant as that of the patient. As an example, cosegregation of variant in the family of Patient RB4 was shown in Figure 2A. Interestingly, a *de novo* heterozygous in-frame deletion of three bases identified at the start site of exon 20 in patient RB24 was considered to be deleterious, which might result in splicing defect (Figure 2B).

**Figure 3:**
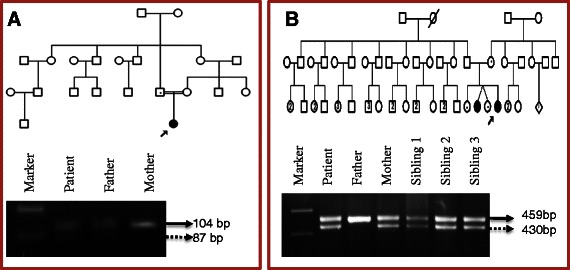
Agarose gel electrophoresis for the confirmation of small deletions. **(A)** 17 bp deletion in the Promoter region was observed in blood samples of patient RB1 and his father. **(B)** 29 bp deletion in Exon 1 was observed in blood samples of patient RB11, her mother and siblings. The size of actual and deleted product is indicated by straight and dotted arrows respectively in both gels.

A heterozygous deletion of 17 bases at the upstream of ORF in the promoter region was detected in patient RB1, which was confirmed by agarose gel electrophoresis. The deletion was also detected in father, who was diagnosed as regressed RB (Figure 3A). In addition, frameshift deletions were detected in three patients. One patient RB15 had deletion of four bases which was also detected in his father. A deletion of 29 bases in exon 1 was identified in patient RB18. In family of patient RB11, a deletion of 29 bases at another locus of exon 1 was observed in all members except father (Figure 3B), where one sibling was affected with RB (Table 1).

### Identification of somatic SNVs and InDels in RB tumor samples

Somatic variants were detected in tumor samples of 7 out of 11 patients with sporadic unilateral RB (Table 1 and Table 3). Homozygous variants were identified in 4 patients (RB10, RB12, RB22 and RB29) and two heterozygous variants were identified in other 3 patients (RB8, RB9 and RB31). Of the homozygous variants, three were nonsense variants in patient RB10, RB12 and RB29, while one novel frameshift variant was identified in Patient RB22. One nonsense and another splice site variant were identified in both Patients RB8 and RB9, and two nonsense variants were identified in patient RB31. In addition, a somatic loss of heterozygosity (LOH) was detected in tumor sample of Patient RB25, where the germline heterozygous nonsense variant (c.1072C > T) was converted to homozygous (Table 2). All the somatic variants and zygosity were confirmed by Sanger sequencing in patient tumor and blood samples. Our results are consistent with the Knudson’s two hit hypothesis [28] in all the patients as we have identified either homozygous or two heterozygous variants.

**Table 3:** **RB1 variants identified by targeted NGS in tumor samples of RB patient**

Patient no	cDNA change	Amino acid change	Functional consequence
RB9	c.380G > A/c.1363C > T	p.S127N/p.R455X	Missense-Altered splicing/Premature Protein Termination
RB10	c.763C > T*	p.R255X*	Premature Protein Termination
RB12	c. 1072C > T*	p.R358X*	Premature Protein Termination
RB22	c.1732_1733delGinsTT*	p.D578LfsX6 *	Frameshift
RB29	c.1654C > T*	p.R552X*	Premature Protein Termination
RB31	c.409 G > T/c.751 C > T	p.E137X/p.R251X	Premature Protein Termination Premature Protein Termination

Novel variant is marked in bold. In patients RB10, RB12, RB22 and RB29, homozygous variants (marked with *) were identified. All the variants given in the table were somatic variants as they were detected only in patient’s tumor but not in blood samples of patient and family members.

### Detection of copy number variations (CNVs)

**Figure 4:**
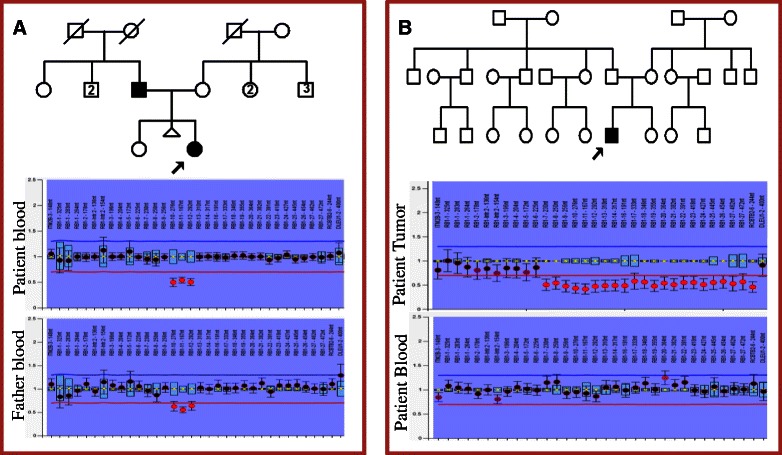
Confirmation of copy number variations (CNVs) by MLPA. **(A)** Patient RB7 had an affected father and both of them showed deletion of exons 10-12. **(B)** Patient RB32 had a somatic deletion of exons 7-27 which was not detected in blood. The deletions were denoted by the red spots below the deletion cut-off line (red) in the ratio chart.

Eleven samples with no pathogenic SNVs and InDels were subjected to the analysis of CNVs. For blood samples, we utilized the tool Cn. MOPS, which detected five heterozygous germline CNVs in four samples. Deletion found in each patient sample RB3, RB5, RB6, RB7 was confirmed by MLPA (Table 4). Of those, deletion of exon 10-12 in patient RB7 cosegregated with phenotype (Figure 4A). Another deletion (exon 22) in patient RB6 detected by Cn. MOPS, was not found by MLPA. Somatic deletions including a homozygous deletion of Exon10 in patient RB21 and a heterozygous deletion of Exons 7-27 in patient RB32 (Figure 4B) were observed using ExomeCNV, which were further confirmed by MLPA (Table 4). Overall, 80% and 100% sensitivity were observed in detecting germline and somatic CNVs respectively.

**Table 4:** **Copy number variations (CNVs) identified in tumor/blood samples of Retinoblastoma patients**

Patient no	CNV	logR	Method used	Cosegregation in family	MLPA confirmation
RB3	Deletion of whole RB1	-1.0	Cn. MOPS	*De novo*	Yes
RB5	Deletion of exons 4-6	-1.0	Cn. MOPS	*De novo*	Yes
RB6	Deletion of exon 22	-1.0	Cn. MOPS	-	No
RB6	Deletion of exons 24-25	-5.5	Cn. MOPS	Heterozygous Father	Yes
RB7	Deletion of exons 10-12	-1.2	Cn. MOPS	Heterozygous Father	Yes
RB21	Deletion of exon 10	-5.4	ExomeCNV	-	Yes
RB32	Deletion of exons 7-27	-1.1	ExomeCNV	-	Yes

Two programs, Cn. MOPS and ExomeCNV were used to identify germline and somatic CNVs from blood and tumor samples respectively. CNVs identified were confirmed and cosegregation was observed by MLPA. The exon 22 deletion identified in patient RB6 was not detected by MLPA. Except the exon 10 homozygous deletion identified in patient RB21, all other CNVs were heterozygous.

### Cross platform comparison of Illumina Miseq and ion-torrent PGM results

The five pathogenic variants detected in patients RB2, RB4, RB13, RB24 and RB25 (Table 2) were concordant with Ion PGM results. Both platforms detected somatic variants in tumor samples (Table 3) and their absence in blood samples of same patients (RB8 and RB12). However, deletion found in patient RB7 (Table 4) was not detected by Ion Torrent Suite. Further, analysis by Cn. Mops could not be carried out because of small sample size.

### Unsolved cases

No pathogenic variants were detected in five patients (RB20, RB23, RB28, RB30 and RB33) with our approach. Rare variants not following our criteria for pathogenicity and deep intronic variant were excluded. For example, in two unsolved cases (RB20 and RB23) one missense variant (Exon19, c.A1846G, p.K616E) was detected. Although it was reported in Human Gene Mutation Database (HGMD) [29], it was not predicted as pathogenic with SIFT, Polyphen2 and MutationTaster tools. It was also observed in more than 12 patients with low coverage and not detected with Sanger sequencing. In another example, one deep intronic variant (exon 3, c.380 + 150 T > A), reported in COSMIC database (ID = COSM164493) and detected in patient RB30, RB23 and RB33 was excluded from the analysis. Moreover it was found to be polymorphism in dbSNP and observed in more than 80% of our patient samples.

## Discussion

Retinoblastoma, the most common childhood intraocular tumor has complex genetic basis of cancer development, initiated by biallelic inactivation of *RB1* gene [28]. Genetic testing of *RB1* will be beneficial to provide counselling for families. However, genetic analysis of heterogeneous spectrum of variants in *RB1* gene is no trivial task [4] and essentially requires comprehensive approach. Here, we have used NGS approach for the molecular analysis of Indian patients with RB, based on *RB1* gene target enrichment, multiplexing and bioinformatics pipeline. We used in-house pipeline to successfully detect both pathogenic germline and somatic variants in RB patients. With our approach, we were able to identify heterogeneous spectrum of *RB1* gene variants including SNVs, InDels and CNVs. All the variants detected were validated using Sanger sequencing, MLPA and size fractionation methods. Thus, our approach, achieving a diagnostic rate of 85%, proved to be efficient for the molecular diagnosis of RB. Moreover, the cross platform comparison with Ion-Torrent PGM results further confirmed the efficiency of NGS.

An important consideration about NGS for diagnosis is identifying the pathogenic variants among the large number of variants detected. In order to identify pathogenic variants, we used stringent criteria after several modifications during the pipeline development. The filtering process has been set to include synonymous and polymorphic variants as potential variants if they are present in cancer and disease databases. While those not present in any databases were classified as rare variants. By applying stringent criteria, we could detect known and novel pathogenic variants with no false positives. For example, a novel intronic variant (c.265-9 T > A) in patient RB4 (Figure 2A) creates a cryptic splice site and is most likely a pathogenic variant. We further confirmed its pathogenicity by cosegregation with phenotype. However, another splice variant (c.1961_1963del) in patient RB24 (Figure 2B) as predicted as most likely pathogenic did not co-segregate with phenotype. Ultimately, functional studies are necessary for assigning pathogenicity to these novel variants.

The limitation of the targeted NGS is the uneven capture efficiency that reduced the sensitivity of detection of CNVs. The capture efficiency was highly variable with the library prepared with Illumina-Truseq and also there were no coverage of exon 14 and few regions of exon 20. This drawback was overcome with the Agilent Sureselect method. However, variable depth of coverage was noted in exons 1 and 27 (10-200X). Hence uniform capture efficiency with a higher depth of RB1 sequencing will resolve the issues.

In addition to the technical limitation of the targeted NGS, complete RB1 sequencing is needed to detect the missed variants in the deep intronic and untranslated regions (UTRs) that could possibly reduce the five unsolved cases. However, there are other factors that can initiate RB, such as promoter methylation of *RB1* gene, and MYCN gene amplification [30]. In fact, we found MYCN amplification in tumor sample of a unilateral patient RB30 (data not shown). Hence, we propose that NGS panel for RB should include MYCN gene along with *RB1*.

Overall, targeted NGS approach is becoming more feasible and efficient in clinical settings, especially for cancer and can potentially identify germline and somatic variants comprehensively. However, we still suggest conventional methods for validation of the variants as we are in the initial phase of developing NGS methods for the diagnosis of RB. Further studies are necessary for the establishment of this approach in terms of cost-effectiveness.

## Conclusions

This is the first such study (to the best of our knowledge) using multiplexed targeted NGS approach to detect pathogenic variants in the *RB1* gene. We reported here that this approach with bioinformatics pipeline could detect germline and somatic variants including novel pathogenic variants. We demonstrated for the first time that this approach could detect copy number variations (CNVs) in *RB1* gene. This comprehensive approach reduces the time and number of assays required for the detection of pathogenic variants by conventional methods. Our approach is sensitive (0.97) and efficient for *RB1* screening.

## Abbreviations

RB: Retinoblastoma
SNV: Single nucleotide variant
InDel: Small insertions/deletions
CNV: Copy number variation
LOH: Loss of heterozygosity
MLPA: Multiplex ligation-dependent probe amplification
NGS: Next generation sequencing
HGMD: Human gene mutation database
COSMIC: Catalogue of somatic mutations in cancer
dbSNP: Database for single nucleotide polymorphisms
ESP: Exome sequencing project
